# Transcriptome-wide mapping reveals a diverse dihydrouridine landscape including mRNA

**DOI:** 10.1371/journal.pbio.3001622

**Published:** 2022-05-24

**Authors:** Austin S. Draycott, Cassandra Schaening-Burgos, Maria F. Rojas-Duran, Loren Wilson, Leonard Schärfen, Karla M. Neugebauer, Sigrid Nachtergaele, Wendy V. Gilbert

**Affiliations:** 1 Yale School of Medicine, Department of Molecular Biophysics & Biochemistry, New Haven, Connecticut, United States of America; 2 Massachusetts Institute of Technology, Department of Biology, Cambridge, Massachusetts, United States of America; 3 Yale University, Department of Molecular, Cellular, and Developmental Biology, New Haven, Connecticut, United States of America; Johns Hopkins University, UNITED STATES

## Abstract

Dihydrouridine is a modified nucleotide universally present in tRNAs, but the complete dihydrouridine landscape is unknown in any organism. We introduce dihydrouridine sequencing (D-seq) for transcriptome-wide mapping of D with single-nucleotide resolution and use it to uncover novel classes of dihydrouridine-containing RNA in yeast which include mRNA and small nucleolar RNA (snoRNA). The novel D sites are concentrated in conserved stem-loop regions consistent with a role for D in folding many functional RNA structures. We demonstrate dihydrouridine synthase (DUS)-dependent changes in splicing of a D-containing pre-mRNA in cells and show that D-modified mRNAs can be efficiently translated by eukaryotic ribosomes in vitro. This work establishes D as a new functional component of the mRNA epitranscriptome and paves the way for identifying the RNA targets of multiple DUS enzymes that are dysregulated in human disease.

## Abstract 2

This study presents dihydrouridine sequencing (D-seq) for transcriptome-wide mapping of this RNA modification with single-nucleotide resolution, revealing dihydrouridine at new locations across the yeast transcriptome that suggest a broad role in folding functional RNA structures.

## Introduction

Dihydrouridine (D) is a modified version of uridine that is installed by dihydrouridine synthase (DUS) enzymes in all domains of life. It is of great interest to determine the locations of D modifications because elevated expression of DUS and elevated D levels in tumors are associated with worse outcomes for patients in lung [1], liver [2], and kidney [3,4] cancer. DUS target sites in tRNAs are best characterized in budding yeast [5,6] and include multiple positions within the eponymous D loop as well as sites in the variable loops of some tRNAs. D has also been detected in the genomic RNA of Dengue, Zika, Hepatitis C, and Polio viruses [7], but the specific locations are unknown. It is likely that DUS modify additional classes of cellular RNA as recently discovered for other tRNA-modifying enzymes [8]. Notably, DUS1 and DUS3 cross-link to mRNA in both yeast and human cells [9,10] suggesting their potential to modify mRNA target sites.

The D modification is a reduction of the C5-C6 double bond in uridine that has multiple effects on RNA structure. First, D subtly distorts the pyrimidine ring [11] causing destacking of bases in oligonucleotides [12]. D also disrupts the orientation of N3 and O4 in the pyrimidine ring, weakening Watson–Crick base pairing, which likely contributes to the 3 to 5°C reduction in melting temperature of RNA duplexes containing a D [13]. More significantly, D substantially destabilizes the typical C3′-endo conformation of the ribose thereby favoring the C2′-endo conformation in a D nucleotide by 5.3 kcal/mol and in the nucleotide 5′ of D by 3.6 kcal/mol [12]. These changes to the RNA backbone conformation strongly disfavor RNA helical geometry [14] and allow for greater flexibility in RNAs. NMR studies of modified and unmodified versions of the tRNA D loop illustrate the consequences of this effect for RNA folding: The unmodified D loop adopts several conformations that rapidly interconvert, whereas the modified RNA folds into a hairpin with a stable stem and the D in a flexible loop region [15]. Thus, dihydrouridylation of RNA is expected to have large effects on RNA structure.

Profound alteration of RNA conformation and structure by D would be expected to affect multiple steps in mRNA metabolism depending on the location of the D nucleotide. For example, D antagonizes formation of RNA duplexes [13,14], which are required for pre-mRNA splicing (due to base-pairing between splice sites and U1, U2, and U6 small nuclear RNAs (snRNAs)) and for regulation by micro RNAs (miRNAs) (due to base-pairing between target mRNA and miRNA). Intramolecular RNA secondary structures have been found to affect the efficiency and regulation of translation initiation, alternative splicing, RNA localization, and RNA stability (reviewed in [16,17]). D is also expected to stabilize binding of numerous regulatory RNA-binding proteins by favoring the C2′-endo conformation that is preferentially bound by K homology (KH) domains and RNA recognition motifs (RRMs) [18]. KH and RRM domains are responsible for sequence-specific binding by proteins that regulate all aspects of mRNA processing and function.

In this paper, we report the development of a novel method to map D residues in RNA in high-throughput. Our method takes advantage of known D-selective chemistry [19–22] to reduce D and induce reverse transcriptase (RT) stops 1nt 3′ of Ds. We combine this D-selective chemistry with next-generation sequencing to determine the location of Ds across the yeast transcriptome. D-seq identifies known tRNA D sites and uncovers novel D sites in small nucleolar RNA (snoRNA) and mRNA. These novel D sites occur in conserved stem-loop regions of mRNAs and snoRNAs—and are consistent with a broad function for D in folding functional RNA structures. In support of the potential for dihydrouridine to affect mRNA biogenesis, we demonstrate DUS-dependent changes in splicing of a naturally dihydrouridylated pre-mRNA in cells. Our results establish D as a new component of the mRNA epitranscriptome and show that the D-seq method is broadly applicable to identifying and studying the functions of D.

## Results and discussion

**Fig 1:**
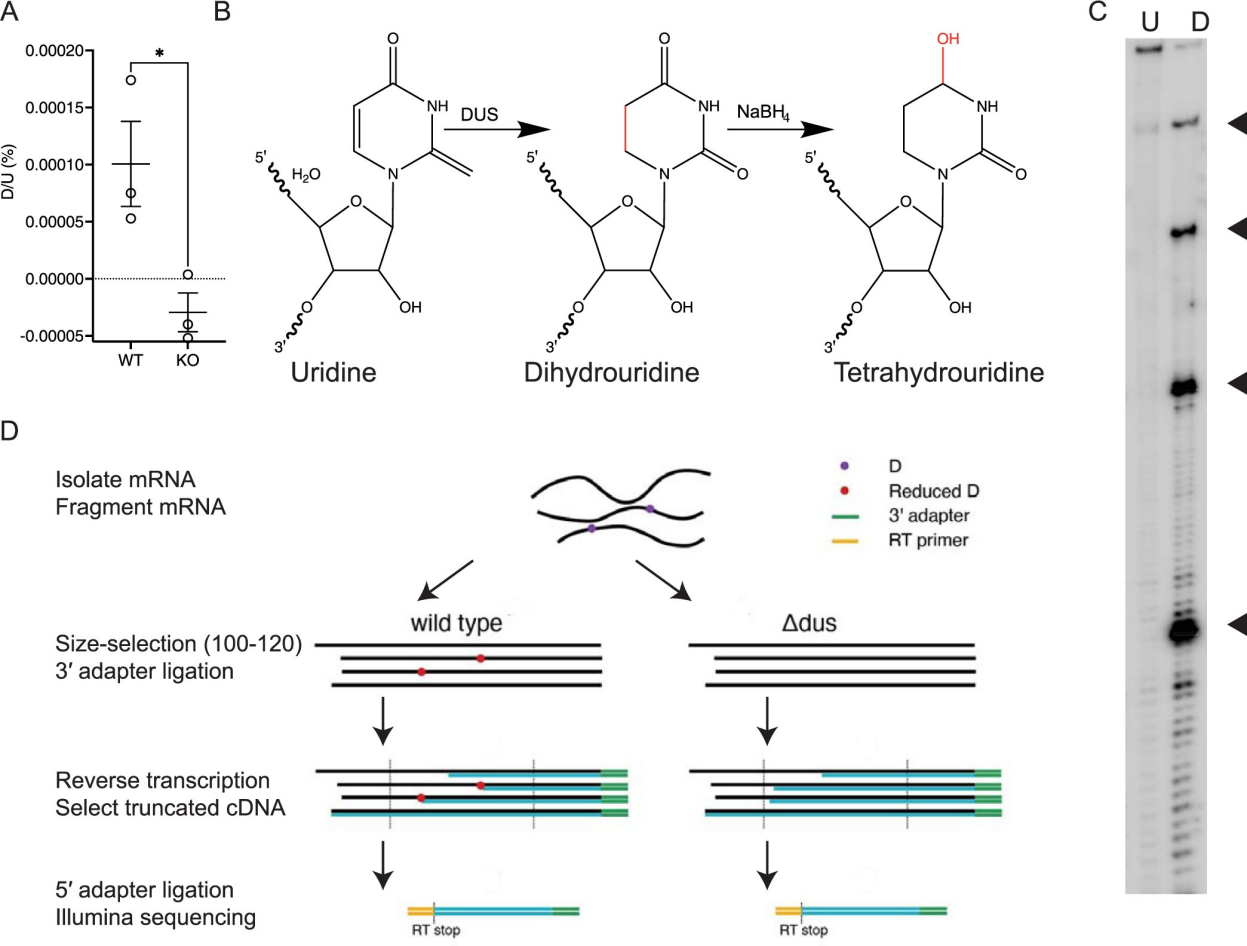
Dihydrouridine-specific chemistry to map dihydrouridine sites in RNA with single-nucleotide resolution. (A) Bulk nucleoside analysis of detects D in mRNA from WT but not DUS KO yeast. mRNA was purified by selecting for poly(A)+ and tRNAs were removed by size selection. (B) Structures of uridine, dihydrouridine, and tetrahydrouridine. (C) Primer extension analysis of synthetic 4D and 4U RNAs treated with NaBH_4_ and reverse transcribed with Super Script III RT. D-dependent RT stop positions are highlighted. (D) Schematic of D-seq library preparation. The data underlying this figure can be found in S3 Table. D, dihydrouridine; D-seq, dihydrouridine sequencing; DUS, dihydrouridine synthase; RT, reverse transcriptase; WT, wild-type.

In light of previous work showing that DUS1 and DUS3 cross-link to mRNA in both yeast and human cells [9,10], we performed bulk nucleotide analysis on RNA from budding yeast. We purified polyA+ mRNA from a *dus1Δ dus2Δ dus3Δ dus4Δ* quadruple mutant strain lacking all DUS activity [6] and a matched wild-type (WT) strain. We detected D in the polyA+ mRNA fraction from WT but not DUS KO (Fig 1A), confirming the hypothesis that DUS enzymes install D in mRNA. We therefore developed a method to map D at single nucleotide resolution by identifying chemical treatments that stall RT at D.

To identify RT stopping conditions for D, we tested different chemistries for selective RT stopping at D compared to U. Strong OH^-^ treatment conditions used previously to map D in tRNA by primer extension [6] proved too harsh to use for mRNA due to substantial RNA degradation (S1A Fig). In contrast, milder sodium borohydride treatment conditions do not damage mRNA-like molecules (S1A Fig). D is selectively reduced to tetrahydrouridine by sodium borohydride to remove a hydrogen bond donor on the Watson–Crick face (Fig 1B) [22]. We prepared 194-nt synthetic RNAs with 4 Us or Ds positioned at approximately 30 nt intervals for easy characterization by primer extension (Methods). Using these RNAs, we found that reduced dihydrouridine blocks several RT enzymes 1 nucleotide 3′ to the D site while having no effect on RT processivity on an identical U-containing template (Figs 1C and S1B). We note that other modified nucleosides not at U can react with sodium borohydride [23]. We combined this D-specific chemistry with strand-specific cDNA sequencing to map the locations of D transcriptome-wide using high-throughput sequencing (Fig 1D).

**Fig 2:**
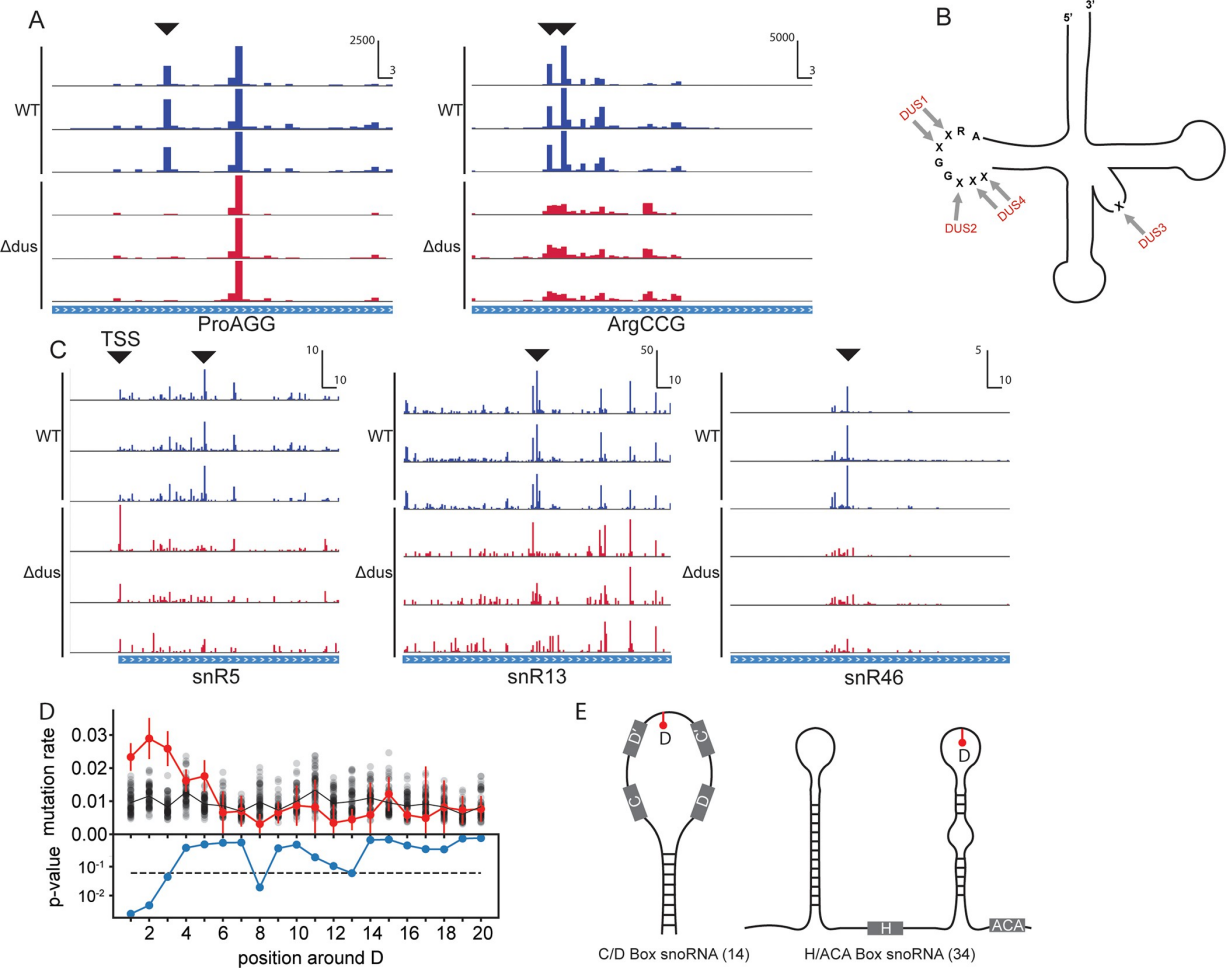
D-seq identifies known and novel dihydrouridine sites in structured ncRNAs. (A) Plots of cDNA end positions in Dus2 target tRNA ProAGG and Dus2, Dus4 target tRNA ArgCCG. D Peaks are highlighted. X scale in RPM and Y scale in bp. (B) Summary of known tRNA D positions and corresponding DUS. (C) Plots of cDNA end positions in snR5, snR13, and snR46 snoRNAs. D peaks are highlighted. TSS (transcription start site) of snR5. X scale in RPM and Y scale in bp. (D) snoRNA Ds occur primarily in stem-loop structures that resemble tRNA D loops. Plot of median DMS-induced mutation rate in 25 nt window flanking D site. Red trace is median DMS reactivity flanking D positions. Black dots are median DMS reactivity for randomly selected set of background positions. Blue trace is *p*-value for difference in DMS reactivity for sequences flanking D or background sites. (E) D sites occur in stem-loop structures of 16 H/ACA and 7 C/D box snoRNAs. The data underlying this figure can be found in S1 and S2 Tables. DMS, dimethyl sulfate; D-seq, dihydrouridine sequencing; ncRNA, non-coding RNA; snoRNA, small nucleolar RNA; TSS, transcription start site.

We tested the D-seq approach in budding yeast where positive control D sites in cytoplasmic tRNAs have been extensively although not exhaustively characterized [5,6]. We observed strong DUS-dependent pileups of cDNA ends 1nt 3′ of many known tRNA D sites (Fig 2A and S1 Table). Given these encouraging findings, we developed a quantitative approach to evaluate D-seq signal by calculating a modified Z-score (MAD score) as a measure of the strength of the RT stop signal at every nucleotide. We used the difference between the distributions of MAD scores at known tRNA D sites (based on previous analysis by micro array and primer extension [6]) in WT and DUS KO libraries (S2A Fig) to set cutoffs for defining a D site in abundant RNAs (Methods). Using these cutoffs, we identified previously reported target sites of 3 of the 4 DUS as well as previously unannotated D sites in 9 tRNAs at positions in the D loop that are known to be modified by DUS1 and DUS4 in other tRNAs (Figs 2A, 2B, and S2B and S1 Table, which compares these sites to previous annotations). We identified a single unanticipated site at U32 in tRNA IleAAT (S1 Table).

As implemented here, D-seq has specific “blind spots” in tRNAs. First, the cDNA size selection step precluded detection of DUS3-dependent Ds at position 47 because they are too close to the 3′ end of the transcript. In addition, several known target sites of DUS1, DUS2, and DUS4 were not detected because they are shadowed by another D 3′ of them (S2B Fig and S1 Table). Other known tRNA D sites that were not visible occur 3′ of a penetrant RT-stop at position 26 in some tRNAs (S2C Fig and S1 Table). We suspect this RT stop is caused by N2,N2-dimethylguanosine (m^2,2^G) [24]. Pretreatment of RNA samples with AlkB demethylases to remove m^2,2^G as well as 1-methyladnosine (m^1^A) and 1-methylguanosine (m^1^G) [25–27] should overcome this limitation. Advantages of the D-seq method are that it inherently offers single-nucleotide resolution and can, in principle, be used to detect D sites in any type of RNA present in the sample.

We then examined other classes of non-coding RNAs (ncRNAs) with sufficient coverage (Methods). We identified 48 novel D sites in 23 different snoRNAs, uncovering snoRNAs as a substantial new class of RNA targeted by DUS enzymes (Fig 2C and S2 Table). We considered the possibility that DUS might modify ribosomal RNA given that dihydrouridine has been reported in the bacterial ribosome at U2449 of the large subunit RNA [28]. However, inspection of the cytoplasmic rRNAs did not reveal any DUS-dependent modification at the orthologous position (S2D Fig).

Like tRNAs, snoRNAs must fold to perform their cellular function [29,30]. Given the importance of D for tRNA folding [12,15], we analyzed chemical probing data to determine if D occurs within structurally stereotyped regions in snoRNAs. Dimethyl sulfate (DMS) methylates the Watson–Crick face of unpaired As and Cs, which can be detected as sites of misincorporation by RT. The observed mutation rate at each A and C indicates the extent of pairing [31], with paired nucleotides having low DMS reactivity and low mutation rates and unpaired loop regions having high reactivity and high mutation rates. Comparing snoRNA D sites with DMS probing data from WT yeast cells [31] revealed a propensity for D to occur in unpaired regions (Fig 2D). Intriguingly, most of the 48 snoRNA D sites are located in 4–8 bp stem-loop regions (schematized in Fig 2E). These compact stem loops are structurally similar to the D loops of tRNAs, suggesting a common mechanism of recognition by DUS and/or a similar role for D within the loop region to promote stable folding of the adjacent stem by causing changes to the RNA backbone conformation [12,15]. Our results establish that DUS modify additional ncRNAs beyond tRNAs and suggest a broad role for DUS in the biogenesis and function of many structured RNAs.

We next analyzed yeast mRNA for D. We used a simple statistical metric, a modified Z-score, to distinguish robust DUS-dependent RT stops from noise in these less abundant RNAs. (See Methods for the advantages and limitations of the MAD score and Z-score metrics). As for tRNAs, we defined empirical thresholds for site calling based on differences in the distributions of scores in WT and DUS KO samples (S3A Fig). Applying conservative cutoffs to the mRNA mapping reads (Methods), we identified 130 high-confidence D sites in mRNAs (S2 Table). To estimate the number of false positives, we inverted the analysis (required high Z-scores in the DUS KO replicates and low Z-scores in WT replicates), which identified 5 false positives for an estimated false discovery rate for D sites in mRNA of 3.8%. Two false positives are understandable as “shadow” peaks downstream of a D (S3B Fig). The number of D sites we identified (130) represents a lower bound for the total number of D sites in yeast mRNA as we surveilled only approximately 1% of the yeast transcriptome that met the coverage threshold in all 6 libraries. These results show that interactions between DUS and mRNA [9,10] result in substantial modification and uncover dihydrouridine as a component of the mRNA epitranscriptome.

**Fig 3:**
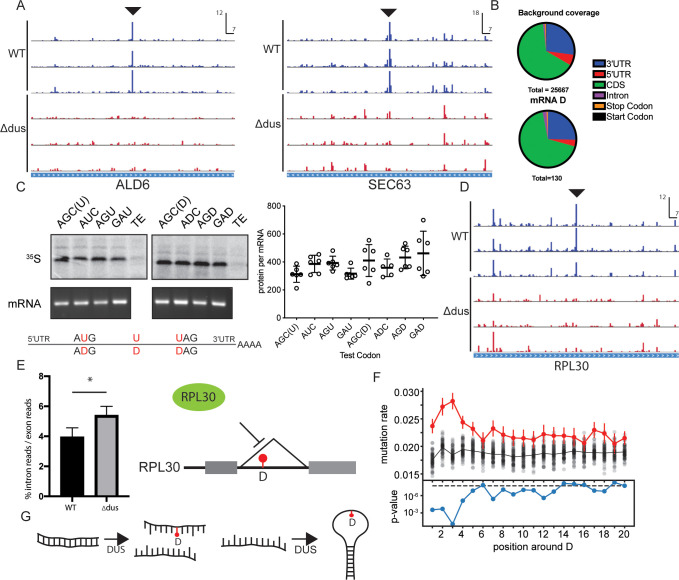
D-seq identifies dihydrouridine sites in mRNAs. (A) Plots of cDNA end positions in ALD6 and SEC63 mRNAs. D peaks are highlighted. Scale in RPM and bp. (B) Distribution of D sites among mRNA features, and background distribution of features for all sites interrogated for D. (C) SDS-PAGE gels showing Top7 protein produced from U and D containing mRNAs with 4 different test codons. Denaturing glyoxal agarose gel showing mRNA integrity. All 4 test constructs showed no significant difference in protein produced per mRNA +/‒ D. Schematic of U/D mRNAs with U/D positions highlighted in red. (D) Plots of cDNA end positions for intronic D in RPL30 mRNA. D peak is highlighted. Scale in RPM and bp. (E) DUS KO strain has increased ratio of RPL30 intron mapping reads to exon mapping reads (*p* < 0.05, Student’s *t* test). Model of regulation of RPL30 pre-mRNA splicing by RPL30 protein. (F) mRNA sequences flanking Ds have higher DMS reactivity indicating greater flexibility. Plot of median DMS-induced mutation rate in 25 nt window flanking D site. Red trace is median DMS reactivity surrounding D positions. Black dots are median DMS reactivity for randomly selected set of background positions. Blue is *p*-value for difference in DMS reactivity for sequences flanking D or background sites. (G) D has multiple impacts on RNA structure. D both promotes loop formation and antagonizes duplex formation. The data underlying this figure can be found in S3 Table. D-seq, dihydrouridine sequencing; DMS, dimethyl sulfate; DUS, dihydrouridine synthase.

The 130 D sites were distributed throughout mRNA features including the 5′-UTR, CDS, introns, and 3′-UTR (Fig 3A and 3B and S2 Table). The prevalence of D in coding sequences, including of essential genes, raised the question of how the presence of D in mRNA impacts translation. We generated model mRNAs encoding a short (12kD) protein, Top7 [32], that can be produced with few uridines: 2 or 3, including the start/AUG, stop/UAG and an internal test codon (Fig 3C). We synthesized mRNAs with no internal U/D test codon, or 1 of 3 different internal codons that we detected as frequently D-modified in endogenous yeast mRNAs, ADC, AGD, and GAD. We translated the D or U versions of these mRNAs in rabbit reticulocyte lysate (RRL) and quantified protein production by measuring ^35^S-Met incorporation into full-length Top7 protein by SDS-PAGE and autoradiography (Figs 3C and S3C). All 8 mRNAs were efficiently translated in RRL with no significant differences in the amount of protein produced from any D or U containing mRNA (*n* = 6 replicates, Figs 3C and S3C). Thus, eukaryotic ribosomes can efficiently traverse D sites in mRNAs. While our results show that the translational output is not impaired by these D-containing codons, other codons may behave differently. It is also possible that D could impact translational fidelity, as has been reported for pseudouridine [33].

In light of the impacts of D on RNA structure [12,13,15], the location of D in the intron of RPL30 (Fig 3D) is notable; this intronic D is adjacent to an RNA structure that is important for the autoregulation of pre-mRNA splicing by free Rpl30 protein [34]. To investigate the potential consequences of this D site for splicing, we performed RNA-seq on WT and DUS KO. The absence of DUS activity caused a reproducible accumulation of unspliced RPL30 transcripts in DUS KO cells that is consistent with a positive effect of D on splicing of this pre-mRNA (Fig 3E). Other D-containing introns (RPL16B and COF1) were not affected indicating that splicing is not generally impaired in the absence of DUS activity (S3D Fig).

It is interesting that several additional mRNA D sites occur in regions where secondary structure potential is evolutionarily conserved [35], suggesting biological function for these structures. Although the predicted structures of D sites in mRNA are more diverse than in snoRNAs, 19 of the 130 identified mRNA D sites occurred in structures very similar to the tRNA D-loop, which is consistent with modification of mRNAs at structurally stereotyped positions analogous to previously known D sites in tRNAs. Globally, our analysis of DMS structure-probing data [31] found that mRNA regions flanking D sites were significantly likelier to be unpaired in cells than a background set of sites (*p* < 0.05, Fig 3F). This might be a consequence of modification because D antagonizes RNA duplex formation, and promotes the formation of stem-loop structures [12,13,15] (Fig 3G). Alternatively, accessibility could be important for modification by DUS.

While our manuscript was in review, Finet and colleagues [36] reported the development of a method similar to D-seq, Rho-seq (so named for the coupling of rhodamine to reduced dihydrouridine). They identified sparse D modification of mRNAs from human cells and *Schizosaccharomyces pombe* similar to the frequency of mRNA D sites that we uncovered in *Saccharomyces cerevisiae*. One notable difference between the studies is that Finet and collegues report modest reductions in translation of D-containing mRNAs in vitro for several D-containing codons, including GAD. Our results do not confirm this reported translational defect (Fig 3C). Conceivably, the source of translation components (rabbit reticulocytes versus wheat germ) and/or differences in the mRNA context, including sequences flanking the GAD codons, affect the amount of protein produced.

Our results establish D-seq as a high-throughput method to map dihydrouridine sites with single-nucleotide resolution and reveal new classes of RNA targets for conserved DUS enzymes, which we now show include mRNA. The discovery of D in mRNA validates the function of DUS–mRNA interactions that have been observed from yeast to human cells [9,10]. The D-seq method is broadly applicable to reveal the specific locations of D, including in pathogenic RNA viruses where dihydrouridine has been detected by MS (mass spectrometry) [7] and in tumors where elevated DUS expression is linked to worse patient outcomes [1–4]

## Methods

### Synthetic RNAs for RNA degradation and RT stop testing

Synthetic 100% uridine or dihydrouridine containing RNA (5′-ggaacagaaacagagaaaggaacagagaaagacaU/DaaacagaaagagacaagaacagagacaagaacagU/DggcaggaacagagacaaacagagacaggaacaaU/DgacaggaacagaaagaaacagagacaagcacU/Dcgggcaccaaggacacgaaccggaacgcggaaccaaacgggcaacggaccggac-3′) was generated by run off transcription with T7 RNA polymerase and gel purified on an 8% urea-TBE polyacrylamide gel electrophoresis (PAGE) gel. To compare the harshness of the different D-modifying treatments, a synthetic RNA was incubated either under published D mapping conditions [6], under published D reduction conditions [21,22] or similar D reduction conditions with NaBH_3_CN substituted for NaBH_4_. To measure RT at reduced dihydrouridine, we reverse transcribed reduced U or D RNA with Superscript III RT, using manufacturer conditions. Samples were prepared and run on sequencing gels as in [37].

### Strain construction

Quadruple DUS mutant strain was generated by mating double-knockout strains FX-34 (*dus1Δ dus2Δ)* and FX-42(*dus4Δ dus3Δ)*, followed by sporulation on SD media and subsequent tetrad dissection. Genotyping was confirmed by PCR using the following oligos: oAD56_DUS1kanR_F, GCAAGGTGATCGTCAAACTGCACT, oAD57_DUS1kanR_R, ATGGAGACGGAGTTGAACATTTTCT, oAD58_DUS2kanR_F, TAGAGACGTAGTTATCCATTCGTCC, oAD59_DUS2kanR_R, CTTTGGACGATAAACTAAAGGGTTT, oAD60_DUS3kanR_F, GGTAATAGTACACGGGATGAAGAGA, oAD61_DUS3kanR_R, TATTTTGATTTTCTTGGAACCCATA, oAD62_DUS4kanR_F, ACTGCATTCATTTTTGTTAGAAAGG, oAD63_DUS4kanR_R, CAAGCTATCTGGAAAAGAGGTGTTA.

### RNA isolation, poly-A selection, and validation

Yeast total RNA was isolated by hot acid phenol extraction from 750 mL OD 6 culture, followed by isopropanol precipitation. Poly-A RNA was isolated from 8 mg total RNA using oligo dT cellulose beads (NEB, Ipswich, MA), as described [38]. Two sequential rounds of poly-A selection were performed. For analysis of mRNA by liquid chromatography–mass spectrometry (LC–MS)/MS, residual tRNAs were removed by size selection (>200 nt) on a zymo RNA Clean & Concentrator column according to manufacturer instructions. Removal of small RNA and ribosomal RNA contamination was verified by automated gel electrophoresis using an Agilent Bioanalyzer 2100.

### D-seq library preparation

Yeast RNA was fragmented in 10-mM ZnCl2 at 94°C for 1 minute and precipitated. BH_4_ treatment of RNA was as follows: Poly-A+ mRNA was resuspended in 18 μL ddH2O and treated 2 μL of 10 mg/mL NaBH_4_ in 500 mM Tris pH 7.5 at 0°C for 1 hour. The BH_4_ treatment was quenched with 4 μL of 6 N CH_3_COOH and precipitated. RNA fragments were dephosphorylated and end repaired with CIP (NEB) and PNK (NEB), followed by size selection of RNA fragments (70 to 80 nt) on an 8% urea-TBE PAGE gel. RNA fragments were eluted from gel slices overnight at 4°C with gentle rocking in 400 μL RNA elution buffer (300 mM NaOAc pH 5.5, 1 mM EDTA, 100 U/ml RNasin (Promega, Madison, WI, USA) followed by precipitation). Ligation of a preadenylated adaptor (IDT) was carried out with T4 RNA ligase (NEB) in buffer without ATP (50 mM Tris-HCl pH 7.8, 10 mM MgCl2, 10 mM DTT) at 22°C for 2.5 hours, followed by precipitation. Adapter ligated RNA fragments were reverse transcribed with SuperScript III RT (Thermo, Waltham, MA). RNA and primer were denatured and annealed at 95°C for 5 minutes then placed on ice for 5 minutes. After annealing, 1 μL 10 mM dNTPs, 1 μL 0.1 M DTT, 1 μL RNAsin (Promega), 3 μL 5× First Strand Buffer (Thermo), 1 μL SSIII RT were added, and reverse transcription was carried out at 50°C for 1 hour. Truncated cDNAs were size selected (50 to 80 nt) and purified on an 8% urea-TBE PAGE gel, followed by precipitation. cDNAs were eluted from gel slices overnight at room temperature with gentle rocking in 400 μl DNA elution buffer (300 mM NaCl, 10 mM Tris, pH 8.0). A 5′ adapter was ligated on to the cDNA using T4 RNA ligase. 0.8 μL 80 uM linker (IDT) was mixed with 1 μL DMSO, 5 μL of eluted cDNA, incubated at 75°C for 2 minutes and immediate placed on ice for 2 minutes. After cooling, 2 uL RNA Ligase buffer (NEB), 0.2 μL.1 M ATP (NEB), 6.5 μL PEG-8000 (NEB), 3.6 μL ddH2o, and 0.5 μL T4 RNA ligase 2 were added to the cDNA/linker mix. The ligation was incubated overnight at 22°C. The ligation was cleaned up with Dynabead MyOne Silane magnetic beads (Thermo) according to the manufacturer’s instructions. Sequencing libraries were amplified from 5′ and 3′ linker ligated cDNA using Phusion DNA polymerase (NEB). PCR products were gel-purified, precipitated, pooled, and sequenced on an Illumina HiSeq 2500.

### RNA-seq library preparation

RNA seq libraries were generated in parallel with D-seq libraries by omitting the NaBH_4_ treatment and selecting for full length (90 to 100 nt) RT products.

### Sequencing data analysis

Demultiplexed reads were adapter trimmed using BBTools [39,40] bbduk.sh. Adapter-trimmed reads were then PCR-duplicated collapsed based on unique molecular identifier (UMI) using dedupe.sh. The UMI was then force trimmed with a second round of trimming. Adapter trimmed and duplicate collapsed reads were then aligned to the sacCer3 genome using bbmap.sh. For the tRNA mapping D analysis, we aligned the trimmed and PCR-duplicate collapsed reads to a pseudo-genome containing 1 copy of each unique tRNA sequence. Uniquely mapping strand-specific read end position was obtained using bedtools [41]. We generated wig files and visualized the read end positions using mochiview [42]. D peaks were annotated using the sacCer3 features file and bedtools.

### mRNA D-seq peak calling

D-sites were identified as statistical outliers in position-specific accumulation of D-seq reads. We required at least 50 reads in the 100 nt window surrounding the test position and quantified and compared sites using a modified Z-score, in which the position of interest is excluded when calculating the mean and standard deviation. For all of the test positions that met the read cutoff, we calculated a Z-score of read ends based on the distribution of read ends in the 100 nt window centered on the test position.


$$Z_{pos}=\frac{{ends}_{pos}-mean({ends}_{window})}{stdev({ends}_{window})}$$

### tRNA and snRNA D-seq peak calling

In the highly structured and heavily modified tRNAs and snRNAs, we identified D sites by analyzing the absolute deviation around the median (MAD), which scores sites relative to the median rather than the mean. See below regarding the choice of metric for different classes of RNA. We considered every position in the test transcriptome where there were more than 50 reads in the 100 nt window surrounding the test position and additionally required that the window median was greater than zero. For all test positions that met the read cutoff, we calculated a MAD score of read ends based on the distribution of read ends in the 100 nt window centered on the test position.


$$M_{pos}=\frac{{ends}_{pos}-median({ends}_{window})}{MAD({ends}_{window})}$$

And:

$$MAD=median(|{ends}_{pos}-median({ends}_{window}|)$$

### Selection of D-seq signal scoring metrics

Two different scoring approaches were necessary for unbiased identification of D sites in different classes of RNA due to large differences in the distributions of strong RT stops as well as RNA abundance. The presence of multiple RT stops in close proximity, which is common in tRNA due to pervasive RNA modification and strong secondary structure, precludes use of the intuitive and statistically principled Z-score which we use for less structured mRNAs. The Z-score cannot identify RT stop signals in windows with multiple strong signals because these background RT stops in ncRNA dampen the signal at the position of interest [43] (S2E Fig). We therefore analyzed ncRNAs using the absolute deviation around the median (MAD), which scores sites relative to the median rather than the mean. The MAD score requires much higher coverage than the Z-score because, for the denominator to be non-zero, more than half the positions in the window surrounding a site must have 5′ ends mapped to them. In mRNA, it is rare for a 100-nt window to contain more than 1 strong RT stop and the modified Z-score is preferred to quantify and compare sites.

### D-seq peak thresholding

For each set of peaks (tRNA, snRNA, mRNA), we defined library cutoffs for peak score by plotting the distribution of peak scores in each library as an inverse CDF. We set cutoffs for D peaks by requiring D site scores be substantially greater than the score at which the distributions of WT and DUS KO diverge in 3 out of 3 WT replicates, and less than the score at which the distributions diverge in the quad KO. For tRNA, MAD score cutoffs were MAD_wt_ > 40 and MAD_dus_ < 40. For snRNA, MAD score cutoffs were MAD_wt_ > 12 and MAD_dus_ < 8. For mRNA, the Z-score cutoffs were Z_wt_ > 10 and Z_dus_ < 7.

### DMS reactivity near D sites

A previously published DMS-MaPseq [31] data set for *S*. *cerevisiae* was downloaded from GEO (accession number GSE84537). Raw reads were preprocessed and aligned according to the original publication, and DMS reactivities were calculated as the ratio of mutations to coverage. Reactivities around called D sites in mRNAs or snRNAs were pooled for each nucleotide position relative to the respective D coordinate. Values were included in the analysis only if the coverage was larger than 350 and the nucleotide identity in the transcript was A or C. To visualize background reactivities and control for nucleotide bias, 70 samples matching the size of called D sites were randomly drawn from a population of background sites that fell below the Z-score cutoffs in WT and quad KO. Significance testing was done using 2-sided Mann–Whitney U tests between reactivities around the full set of background positions and the called D sites.

### Detecting dihydrouridine by LC-MS/MS

For each sample, duplicate digestions of 50 ng and 750 ng for each sample were digested with 5 U/uL Benzonase (Sigma, St. Louis, MO, #E8263), 0.1 U/uL phosphodiesterase (Sigma, #P3243), 1 U/uL alkaline phosphatase (Sigma #P5521) in 500 mM Tris-HCl pH 8.0, 10 mM MgCl_2_ in a final reaction volume of 50 μL for 6 hours at 37°C. An equal volume of water was then added to each sample before filtration through a 0.2 μm PVDF filter (0.2 μm pore size, 0.4 mm diameter, Millipore, Burlington, MA). A total of 10 μL of each sample was separated by reverse phase ultra-performance liquid chromatography on a Shim-pack GIST C18 2 μm, 2.1 × 50 mm column (Shimadzu, Kyoto, Japan, #227-30001-02) on a Nexera LC-40D XS liquid chromatography system using a gradient of 5 mM ammonium acetate pH 5.3 and acetonitrile. After separation, samples were analyzed by mass spectrometry on a Shimadzu LCMS-8060 Triple Quadrupole Liquid Chromatograph Mass Spectrometer (Shimadzu). Nucleosides were quantified using the following nucleoside-to-base transitions: 267.966 > 136.000 (A), 284.004 > 152.100 (G), 245.30 > 113.10 (U), and 247.20 > 115.15 (D). Mixes of nucleoside standards were injected alongside the samples in the same run to generate standard curves, from which concentrations of each nucleoside in each sample were calculated. The percentages of modified to unmodified nucleoside in each sample were calculated based on calibrated concentrations. These conditions were adapted from reference [36].

### Synthesis of U and D substituted mRNAs

mRNAs were designed based on the coding sequence of Top7 [32] by replacing all but 3 uridine-containing codons: start, stop, and a single test codon. The UTRs also lacked U. RNA was synthesized with T7 by runoff transcription of linearized plasmid templates with either 100% UTP or 100% DTP (Trilink Biotechnologies, San Diego, CA) and purified on 6% denaturing Urea-PAGE gels. Samples of purified mRNAs were denatured with glyoxal at 50°C for 30 minutes and analyzed for integrity by separation on 1% agarose gels in BPTE buffer (100 mM PIPES, 300 mM Bis-Tris, 10 mM EDTA, pH 6.5) and imaging (Bio-Rad ChemiDoc, Hercules, California). Bands were quantified using GelAnalyzer 19.1 (www.gelanalyzer.com).

### In vitro translation of U and D substituted mRNAs

Uncapped Top7 mRNAs were translated in nuclease-treated RRL (Promega). The 500 ng mRNA was incubated in 8.4 μL RRL, 0.24 μL 1 mM amino acid mixture minus methionine, 0.48 μL ^35^S methionine, 0.24 μL RNasin, and ddH_2_O to 20 μL. Translation reactions were incubated at 30°C for 90 minutes and quenched with 20 μL 2X SDS sample buffer. Translation reactions were then incubated at 60°C for 20 minutes and resolved on a 14% to 20% SDS-PAGE gel. Gels were fixed in 30% methanol, 10% acetic acid, incubated in Amplify solution (GE Healthcare, Marlborough, MA), and dried on a vacuum drier. Dried gels were exposed for a minimum of 12 hours on a storage phosphor screen (GE Healthcare), scanned (Bio-Rad), and quantified using GelAnalyzer 19.1 (www.gelanalyzer.com).

## Supporting information

S1 Fig Testing RNA treatment and RT conditions for D-seq. (A) Unmodified 194 nt RNA was treated with strong base (OH‒), NaBH_3_CN, or NaBH_4_, precipitated and run on an 8% urea-PAGE gel. (B) Multiple RTs stall at reduced D. 1: 4D RNA treated with borohydride and benzyhydrazide, 1: 4D RNA treated with borohydride 3: 4U RNA 4: 4D RNA. (C) Bioanalyzer analysis of total RNA and PolyA+ mRNA for mass spectrometry. D-seq, dihydrouridine sequencing; RT, reverse transcriptase.
(EPS)

S2 Fig (A) Inverse CDF plots of MAD score for possible D positions (16, 17, 20, 20a, 20b, and 47) in tRNAs, MAD score for all tRNA U positions, and all snRNA U positions. (B). Plots of cDNA end positions in tRNA GlnCTG and tRNA LysTTT containing multiple Ds. The 3′ most D peak is highlighted. (C) Plots of cDNA end positions in SerGCT where D signal is blocked by m^2,2^G. m^2,2^G position is highlighted. (D) Plots of cDNA end positions in cytoplasmic 25S rRNA at the position orthologous to *Escherichia coli* position 2449. The data underlying this figure can be found in S2 and S3 Tables. m^2,2^G, N2,N2-dimethylguanosine; snRNA, small nuclear RNA.
(EPS)

S3 Fig (A). Inverse CDF plots of Z-score for mRNA mapping reads. Blue line is Z_dus_ cutoff and red line is Z_wt_ cutoff. (B) Plots of cDNA end positions for 5 false positive sites in mRNAs. Shadowing D peaks are highlighted in black. False positive sites are highlighted in red. (C) Uncropped Top7 ^35^S SDS-PAGE and mRNA 1% BP-TE agarose gels from Fig 3C. (d) DUS KO strain does not have a change in ratio of intron mapping reads to exon mapping reads for COF1 or RPL16b. The data underlying this figure can be found in S3 Table. DUS, dihydrouridine synthase.
(EPS)

S1 Table Detection of D sites in tRNAs. tRNA name, position, strand, MAD score, standard deviation, and reads in window for each replicate.
(XLSX)

S2 Table Detection of D sites in snRNAs. snRNA name, position, strand, MAD score, standard deviation, and reads in window for each replicate. snRNA, small nuclear RNA.
(XLSX)

S3 Table Detection of D sites in mRNAs. mRNA name, position, strand, Z-score, standard deviation, and reads in window for each replicate.
(XLSX)

S1 Raw images Uncropped Top7 35S SDS-PAGE and mRNA 1% BP-TE agarose gels for both replicates. (PDF)

## Abbreviations

D: dihydrouridine
DMS: dimethyl sulfate
D-seq: dihydrouridine sequencing
DUS: dihydrouridine synthase
KH: K homology domain
LC–MS: liquid chromatography–mass spectrometry
m^1^A: 1-methyladnosine
m^2,2^G, N2: N2-dimethylguanosine
m^7^G: 7-methylguanosine
miRNA: micro RNA
ncRNA: non-coding RNA
RRL: rabbit reticulocyte lysate
RRM: RNA recognition motif domain
RT: reverse transcriptase
snRNA: small nuclear RNA
snoRNA: small nucleolar RNA
UMI: unique molecular identifier
WT: wild-type

## References

[1] Kato T, Daigo Y, Hayama S, Ishikawa N, Yamabuki T, Ito T, et al. A novel human tRNA-dihydrouridine synthase involved in pulmonary carcinogenesis. Cancer Res. 2005;65:5638–46. doi: 10.1158/0008-5472.CAN-05-0600 15994936

[2] Kuchino Y, Borek E. Tumour-specific phenylalanine tRNA contains two supernumerary methylated bases. Nature. 1978;271:126–9. doi: 10.1038/271126a0 202873

[3] Creighton CJ, Morgan M, Gunaratne PH, Wheeler DA, Gibbs RA, Gordon Robertson A, et al. Comprehensive molecular characterization of clear cell renal cell carcinoma. Nature. 2013;499:43–9. doi: 10.1038/nature12222 23792563 PMC3771322

[4] Cancer Genome Atlas Research Network, Linehan WM, Spellman PT, Ricketts CJ, Creighton CJ, Fei SS, et al. Comprehensive Molecular Characterization of Papillary Renal-Cell Carcinoma. N Engl J Med. 2016;374:135–45. doi: 10.1056/NEJMoa1505917 26536169 PMC4775252

[5] Xing F, Martzen MR, Phizicky EM. A conserved family of Saccharomyces cerevisiae synthases effects dihydrouridine modification of tRNA. RNA. 2002;8:370–81. doi: 10.1017/s1355838202029825 12003496 PMC1370258

[6] Xing F, Hiley SL, Hughes TR, Phizicky EM. The Specificities of Four Yeast Dihydrouridine Synthases for Cytoplasmic tRNAs. J Biol Chem. 2004;279:17850–60. doi: 10.1074/jbc.M401221200 14970222

[7] McIntyre W, Netzband R, Bonenfant G, Biegel JM, Miller C, Fuchs G, et al. Positive-sense RNA viruses reveal the complexity and dynamics of the cellular and viral epitranscriptomes during infection. Nucleic Acids Res. 2018;46:5776–91. doi: 10.1093/nar/gky029 29373715 PMC6009648

[8] Roundtree IA, Evans ME, Pan T, He C. Dynamic RNA modifications in gene expression regulation. Cell. 2017;169:1187–200. doi: 10.1016/j.cell.2017.05.045 28622506 PMC5657247

[9] Mitchell SF, Jain S, She M, Parker R. Global analysis of yeast mRNPs. Nat Struct Mol Biol. 2013;20:127–33. doi: 10.1038/nsmb.2468 23222640 PMC3537908

[10] Beckmann BM, Horos R, Fischer B, Castello A, Eichelbaum K, Alleaume A-M, et al. The RNA-binding proteomes from yeast to man harbour conserved enigmRBPs. Nat Commun. 2015;6:10127. doi: 10.1038/ncomms10127 26632259 PMC4686815

[11] Emerson J, Sundaralingam M. Structure of the potassium salt of the modified nucleotide dihydrouridine 3’-monophosphate hemihydrate: correlation between the base pucker and sugar pucker and models for metal interactions with ribonucleic acid loops. Acta Crystallogr B. 1980;36:537–43. doi: 10.1107/S0567740880003780

[12] Dalluge JJ, Hashizume T, Sopchik AE, McCloskey JA, Davis DR. Conformational flexibility in RNA: the role of dihydrouridine. Nucleic Acids Res. 1996;24:1073–9. doi: 10.1093/nar/24.6.1073 8604341 PMC145759

[13] Sipa K, Sochacka E, Kazmierczak-Baranska J, Maszewska M, Janicka M, Nowak G, et al. Effect of base modifications on structure, thermodynamic stability, and gene silencing activity of short interfering RNA. RNA. 2007;13:1301–16. doi: 10.1261/rna.538907 17585051 PMC1924902

[14] Westhof E, Dumas P, Moras D. Restrained refinement of two crystalline forms of yeast aspartic acid and phenylalanine transfer RNA crystals. Acta Crystallogr A. 1988;44 (Pt 2):112–23. 3272146

[15] Dyubankova N, Sochacka E, Kraszewska K, Nawrot B, Herdewijn P, Lescrinier E. Contribution of dihydrouridine in folding of the D-arm in tRNA. Org Biomol Chem. 2015;13:4960–6. doi: 10.1039/c5ob00164a 25815904

[16] Kwok CK, Tang Y, Assmann SM, Bevilacqua PC. The RNA structurome: transcriptome-wide structure probing with next-generation sequencing. Trends Biochem Sci. 2015;40:221–32. doi: 10.1016/j.tibs.2015.02.005 25797096

[17] Lu Z, Chang HY. Decoding the RNA structurome. Curr Opin Struct Biol. 2016;36:142–8. doi: 10.1016/j.sbi.2016.01.007 26923056 PMC4785074

[18] Kligun E, Mandel-Gutfreund Y. The role of RNA conformation in RNA-protein recognition. RNA Biol. 2015;12:720–7. doi: 10.1080/15476286.2015.1040977 25932908 PMC4615831

[19] Cerutti P, Kondo Y, Landis WR, Witkop B. Photoreduction of uridine and reduction of dihydrouridine with sodium borohydride. J Am Chem Soc. 1968;90:771–5. doi: 10.1021/ja01005a039 5640373

[20] Wintermeyer W, Zachau HG. Fluorescent Derivatives of Yeast tRNAPhe. Eur J Biochem. 1979;98:465–75. doi: 10.1111/j.1432-1033.1979.tb13207.x 114393

[21] Pan D, Qin H, Cooperman BS. Synthesis and functional activity of tRNAs labeled with fluorescent hydrazides in the D-loop. RNA. 2009;15:346–54. doi: 10.1261/rna.1257509 19118261 PMC2648706

[22] Kaur J, Raj M, Cooperman BS. Fluorescent labeling of tRNA dihydrouridine residues: Mechanism and distribution. RNA. 2011;17:1393–400. doi: 10.1261/rna.2670811 21628433 PMC3138574

[23] Behm-Ansmant I, Helm M, Motorin Y. Use of Specific Chemical Reagents for Detection of Modified Nucleotides in RNA. J Nucleic Acids. 2011;2011. doi: 10.4061/2011/408053 21716696 PMC3118635

[24] Ellis SR, Morales MJ, Li JM, Hopper AK, Martin NC. Isolation and characterization of the TRM1 locus, a gene essential for the N2,N2-dimethylguanosine modification of both mitochondrial and cytoplasmic tRNA in Saccharomyces cerevisiae. J Biol Chem. 1986;261:9703–9. 2426253

[25] Zheng G, Qin Y, Clark WC, Dai Q, Yi C, He C, et al. Efficient and quantitative high-throughput transfer RNA sequencing. Nat Methods. 2015;12:835–7. doi: 10.1038/nmeth.3478 26214130 PMC4624326

[26] Cozen AE, Quartley E, Holmes AD, Hrabeta-Robinson E, Phizicky EM, Lowe TM. ARM-seq: AlkB-facilitated RNA methylation sequencing reveals a complex landscape of modified tRNA fragments. Nat Methods. 2015;12:879–84. doi: 10.1038/nmeth.3508 26237225 PMC4553111

[27] Dai Q, Zheng G, Schwartz MH, Clark WC, Pan T. Selective Enzymatic Demethylation of N2, N2 -Dimethylguanosine in RNA and Its Application in High-Throughput tRNA Sequencing. Angew Chem Int Ed Engl. 2017;56:5017–20. doi: 10.1002/anie.201700537 28371071 PMC5497677

[28] Kowalak JA, Bruenger E, McCloskey JA. Posttranscriptional Modification of the Central Loop of Domain V in Escherichia coli 23 S Ribosomal RNA (∗). J Biol Chem. 1995;270:17758–64. doi: 10.1074/jbc.270.30.17758 7629075

[29] Watkins NJ, Dickmanns A, Lührmann R. Conserved stem II of the box C/D motif is essential for nucleolar localization and is required, along with the 15.5K protein, for the hierarchical assembly of the box C/D snoRNP. Mol Cell Biol. 2002;22:8342–52. doi: 10.1128/MCB.22.23.8342-8352.2002 12417735 PMC134055

[30] Khanna M, Wu H, Johansson C, Caizergues-Ferrer M, Feigon J. Structural study of the H/ACA snoRNP components Nop10p and the 3′ hairpin of U65 snoRNA. RNA. 2006;12:40–52. doi: 10.1261/rna.2221606 16373493 PMC1370884

[31] Zubradt M, Gupta P, Persad S, Lambowitz AM, Weissman JS, Rouskin S. DMS-MaPseq for genome-wide or targeted RNA structure probing in vivo. Nat Methods. 2017;14:75–82. doi: 10.1038/nmeth.4057 27819661 PMC5508988

[32] Kuhlman B, Dantas G, Ireton GC, Varani G, Stoddard BL, Baker D. Design of a novel globular protein fold with atomic-level accuracy. Science. 2003;302:1364–8. doi: 10.1126/science.1089427 14631033

[33] Eyler DE, Franco MK, Batool Z, Wu MZ, Dubuke ML, Dobosz-Bartoszek M, et al. Pseudouridinylation of mRNA coding sequences alters translation. Proc Natl Acad Sci U S A. 2019;116:23068–74. doi: 10.1073/pnas.1821754116 31672910 PMC6859337

[34] White SA, Hoeger M, Schweppe JJ, Shillingford A, Shipilov V, Zarutskie J. Internal loop mutations in the ribosomal protein L30 binding site of the yeast L30 RNA transcript. RNA. 2004;10:369–77. doi: 10.1261/rna.2159504 14970382 PMC1370932

[35] Rouskin S, Zubradt M, Washietl S, Kellis M, Weissman JS. Genome-wide probing of RNA structure reveals active unfolding of mRNA structures in vivo. Nature. 2014;505:701–5. doi: 10.1038/nature12894 24336214 PMC3966492

[36] Finet O, Yague-Sanz C, Krüger LK, Tran P, Migeot V, Louski M, et al. Transcription-wide mapping of dihydrouridine reveals that mRNA dihydrouridylation is required for meiotic chromosome segregation. Mol Cell. 2021 [cited 14 Jan 2022]. doi: 10.1016/j.molcel.2021.11.003 34798057 PMC8792297

[37] Smola MJ, Rice GM, Busan S, Siegfried NA, Weeks KM. Selective 2′-hydroxyl acylation analyzed by primer extension and mutational profiling (SHAPE-MaP) for direct, versatile, and accurate RNA structure analysis. Nat Protoc. 2015;10:1643–69. doi: 10.1038/nprot.2015.103 26426499 PMC4900152

[38] Carlile TM, Rojas-Duran MF, Gilbert WV. Pseudo-Seq: Genome-Wide Detection of Pseudouridine Modifications in RNA. Methods Enzymol. 2015;560:219–45. doi: 10.1016/bs.mie.2015.03.011 26253973 PMC7945874

[39] Bushnell B. BBMap. In: SourceForge [Internet]. [cited 2021 May 23]. Available from: https://sourceforge.net/projects/bbmap/.

[40] Bushnell B, Rood J, Singer E. BBMerge–Accurate paired shotgun read merging via overlap. PLoS ONE. 2017;12:e0185056. doi: 10.1371/journal.pone.0185056 29073143 PMC5657622

[41] Quinlan AR, Hall IM. BEDTools: a flexible suite of utilities for comparing genomic features. Bioinformatics. 2010;26:841–2. doi: 10.1093/bioinformatics/btq033 20110278 PMC2832824

[42] Homann OR, Johnson AD. MochiView: versatile software for genome browsing and DNA motif analysis. BMC Biol. 2010;8:49. doi: 10.1186/1741-7007-8-49 20409324 PMC2867778

[43] Leys C, Ley C, Klein O, Bernard P, Licata L. Detecting outliers: Do not use standard deviation around the mean, use absolute deviation around the median. J Exp Soc Psychol. 2013;49:764–6. doi: 10.1016/j.jesp.2013.03.013

