# Supplementary figures and images for: Transcriptome-wide mapping reveals a diverse dihydrouridine landscape including mRNA
Source: PLoS Biol. 2022 May 24;20(5):e3001622. doi: 10.1371/journal.pbio.3001622 (PMC9129914; doi:10.1371/journal.pbio.3001622)

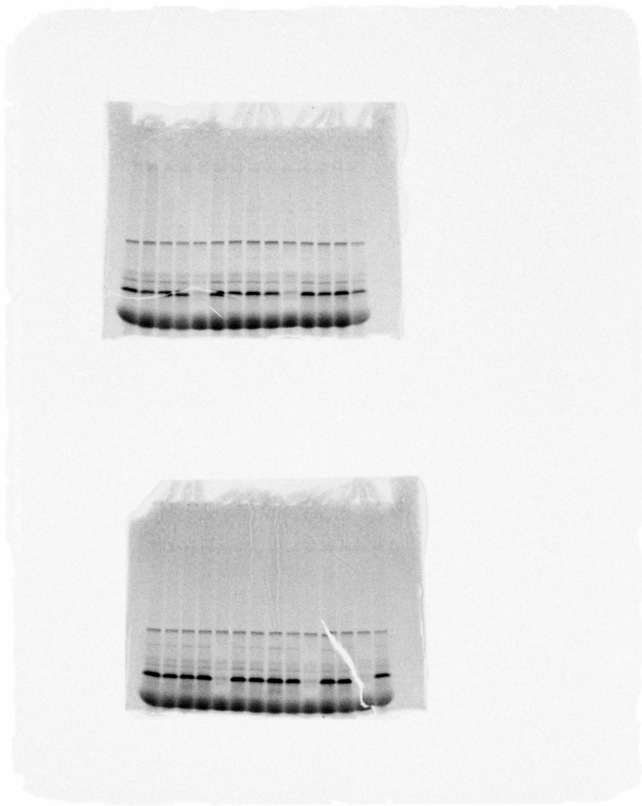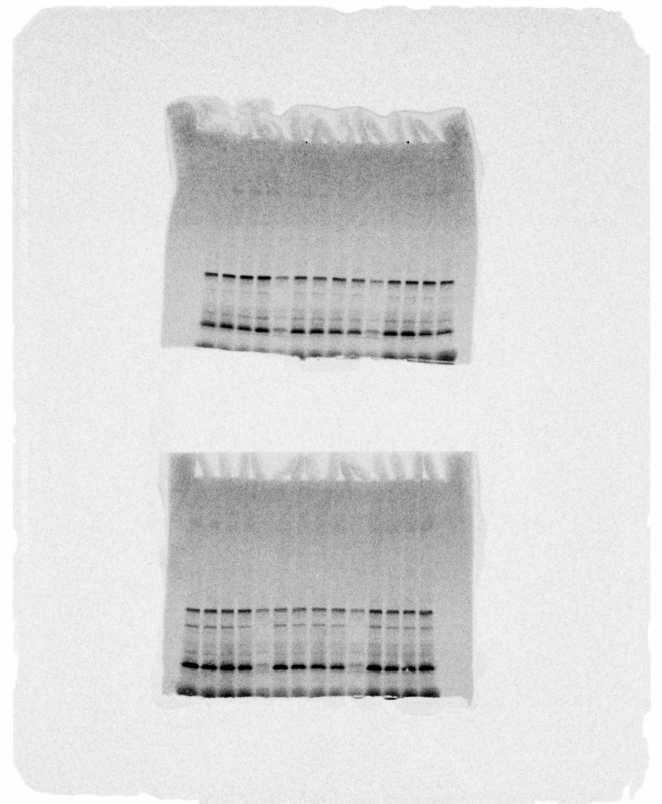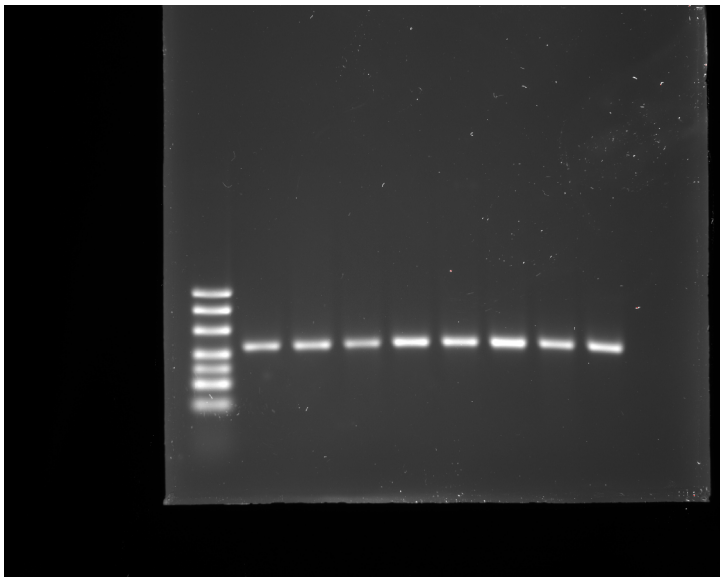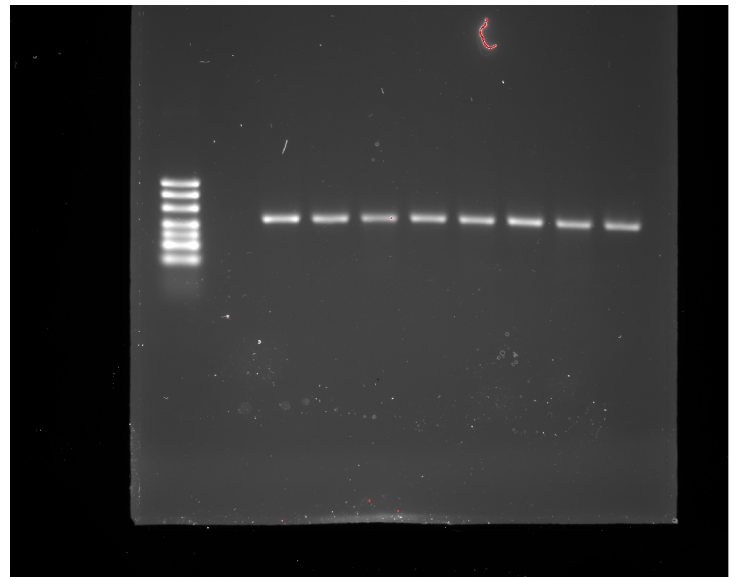

Supplement: S1 Raw images — (PDF) [file pbio.3001622.s007.pdf]
